# Measuring disease likelihood in genomic ascertainment

**DOI:** 10.1016/j.ajhg.2026.03.009

**Published:** 2026-04-07

**Authors:** Julie C. Sapp, Katie L. Lewis, Emily W. Modlin, Alana Davidson, Charlotte Linton Early, Adam H. Buchanan, Alexis Darling, Jacquelyn Mahder, Cara Z. McCormick, Allison J. de Moya, Brooke Rosenblum, Morgan Similuk, Kelly Tangney, Meghan C. Towne, Clesson Turner, Caralynn M. Wilczewski, Jennifer J. Johnston, Leslie G. Biesecker

**Affiliations:** 1 Center for Precision Health Research, National Human Genome Research Institute, National Institutes of Health, Bethesda, MD 20892, USA; 2 Department of Genomic Health, Geisinger, Danville, PA 17822, USA; 3 PWN Health, New York, NY 10011, USA; 4 Centralized Sequencing Program, National Institute of Allergy and Infectious Diseases, National Institutes of Health, Bethesda, MD 20892, USA; 5 Color Health, Burlingame, CA 94010, USA; 6 Ambry Genetics, Aliso Viejo, CA 92656, USA

**Keywords:** secondary findings, genomic screening, genomic ascertainment

## Abstract

Understanding the yield, predictive power, and utility of a secondary finding is critical for policy development and can help inform discussions for population screening. Because American College of Medical Genetics and Genomics (ACMG) Secondary Findings guidelines are applied in diverse testing contexts, we recruited participants from multiple sources to address these questions. We assessed our first 1,500 inquiries to review the disorders/genes that were returned to these individuals. After eligibility screening, we enrolled 227 recipients and completed genotyping, cascade testing, and phenotyping efforts for 163 probands. From evaluating these families, it became clear that there were highly variable outcomes for the diagnostic yield of secondary findings. To objectively and quantitatively assess this, we developed a method to measure the likelihood that the family was, in fact, affected with the disorder associated with the secondary finding variant. We assessed this in detail for 59 families who had a secondary finding of *BRCA1*- or *BRCA2*-related cancer predisposition. Our estimates of the likelihood of a valid clinicomolecular diagnosis ranged from 26.2% to 100%. Over half (51%) of the families met criteria for diagnostic testing, indicating that diagnostic testing for these disorders is underused and that secondary findings testing is being applied inappropriately to these families. These results will be useful for policy refinement for secondary findings and are also relevant to considerations of population genomic screening.

## Teaser

In this study, the likelihood that individuals with medically actionable secondary genomic variants were affected with hereditary cancer ranged from 26.2% to 100%. Over half (51%) met diagnostic criteria for testing, indicating underuse. Assessing the diagnostic yield and utility of secondary findings is crucial to inform policy development for population genomic screening.

## Introduction

A decade after their initial release, much remains unknown about the implementation and clinical impact of the American College of Medical Genetics and Genomics (ACMG) guidelines for medically actionable secondary findings.^1^ While innumerable opinion papers have been written about secondary findings, relatively few publications have described follow-up and evaluation of individuals with such findings.^2^ Several studies have presented results on electronic health record data of individuals with secondary findings,^3^^,^^4^^,^^5^^,^^6^^,^^7^ and others have confirmed that secondary findings can identify individuals at elevated risk of disease susceptibility.^3^^,^^8^^,^^9^^,^^10^^,^^11^^,^^12^

For secondary findings to be converted into improvements in health via intensified screening and preventive care, it is critical to transform secondary findings into more precise risk assessments. Because variant classification is not perfect, some variants currently classified as pathogenic and likely pathogenic are actually benign. Clinical management of individuals who have received secondary findings must account for the selection bias toward unaffected individuals inherent in opportunistic screening. Secondary findings are no different from any screening program; false positives can occur, and it is crucial to identify as many of those as possible. Accounting for testing context and incorporating available family-level genomic and phenotypic data are necessary to correctly assess the clinical utility of secondary findings and provide recipients with the best possible counseling for surveillance and management.

To address these questions, we initiated a longitudinal study of secondary findings recipients to understand several aspects of the process, including diagnostic yield, clinical utility, and determinants of desired precision medicine outcomes, such as receipt of tailored evaluations and surveillance and cascade testing of relatives. To maximize external validity, we broadly recruited individuals with opportunistically identified variants in genes meeting the ACMG criteria for return as secondary findings. In the pilot phase of this study, we developed the processes and infrastructure needed to gather the medical, genomic, family history, and behavioral data needed to address the study aims. The purpose of this report is to describe how we adapted the definition of secondary findings outlined by the ACMG and report preliminary results of the first 1,500 study inquiries regarding enrollment and genes reported and measured the probability of the clinicomolecular diagnoses (CMD)^13^ of individuals with secondary findings.

## Subjects and methods

### Participants and eligibility

This study was approved by the National Institutes of Health institutional review board (IRB) (NCT02595957), and all participants provided written informed consent for the procedures described. Recognizing that the ACMG Secondary Findings guidelines are applied in research studies focused on specific disorders, biobanks enrolling unselected individuals, and consumer-initiated genetic testing in addition to the clinical diagnostic setting, we recruited participants for this study from 41 sources, including individual clinics, health care centers, research studies, support groups, biobanks, clinical and consumer-initiated genetic testing laboratories, and directly through internet marketing (a list of recruitment sources is shown in Table S1).

We screened inquiring and referred individuals to determine their study eligibility. We made at least three contact attempts via phone, email, and/or mail and described non-responders as passive declines if we did not establish contact. To be screen eligible, prospective participants needed to (1) be at least 18 years of age or, if the secondary findings recipient was <18 years of age, a parent or guardian served as their respondent; (2) have received a secondary finding for a gene and phenotype according to the ACMG v.3.1 definition (i.e., that the finding was not primary or diagnostic and determined by the reporting laboratory to be pathogenic or likely pathogenic) at least 4 months prior; and (3) be English or Spanish speaking. As described below, we independently assessed the pathogenicity of the variants. How we evaluated the secondary nature of the finding in the second criterion evolved over time. We discontinued gathering information on a prospective participant when we acquired the first datum that led to an ineligibility determination, and so not all information was collected on all inquiries.

Variant classifications were reviewed from the test report provided by the referral source and/or the prospective participant and re-classified by an ABMGG board certified clinical molecular geneticist (J.J.J.). We used an adaptation of the then most current classification guidelines from ClinGen and the Bayesian points-based adaptation^14^^,^^15^ of the recommendations from Richards et al.^16^

### Procedures

Screen-eligible individuals were assessed for a history of their testing, their relevant medical history (before and after receipt of the finding), their family history, and review of outside medical records when appropriate and available. Each case was reviewed at a team conference where final eligibility determinations were made. In addition to genotypic and phenotypic assessments, we collected social and behavioral data that will be described and reported elsewhere. Only participants with variants in genes recommended for return by the ACMG that we determined to be at least likely pathogenic were eligible for deep phenotyping, cascade testing, and the Bayesian analyses described here. How we assessed the non-diagnostic or secondary (versus primary or diagnostic) nature of each individual’s variant to determine eligibility evolved over time and is outlined below.

When necessary for the study goals and desired by the participants, clinical evaluations at the National Institutes of Health (NIH) Clinical Center were offered. Enrollment, CLIA-valid cascade testing, and phenotyping were also offered to potentially informative relatives for the purpose of determining the presence of a phenotype compatible with the secondary finding in a variant-positive family member. Clinical data obtained from participants and relatives via NIH Clinical Center evaluations were not incorporated into the results we describe here. All study evaluations were offered free of cost to relevant individuals. We investigated relevant family members until we could either identify an individual who harbored the secondary finding genotype and was affected by the relevant phenotype or we had exhausted the family for such candidates. In either case, we considered data collection complete for the family.

We used clinical and genomic data available at intake to retrospectively apply National Comprehensive Cancer Network (NCCN) criteria (v.3.2023) to participants with *BRCA1* (MIM: 113705) and *BRCA2* (MIM: 600185) variants to determine whether they met current testing criteria.^17^ Additionally, we revised our definition of secondary findings to allow for inclusion of participants who had not received results via clinical diagnostic testing and established a two-part definition of secondary findings for precision medicine research.

Statistical comparisons were performed with the χ^2^ test statistic implemented in Excel (Microsoft, Inc.). Confidence intervals were calculated using the modified Wald method (GraphPad Statistics).

### Estimating disease likelihood: The probability of a CMD from a secondary finding

When reviewing families for whom we completed data collection, we were impressed by the range of clinical evidence that would support a CMD^13^ of the condition associated with these genes and recognized a need for a method to quantitatively assess this. We piloted a Bayesian method to estimate the likelihood of disease in an individual who has a secondary finding. Individuals with a variant of <100% pathogenicity may be in one of three states at assessment.

(1) They are affected and manifest the phenotype associated with the variant.
(2) They have a pathogenic variant and are apparently unaffected; they have increased susceptibility and does not manifest the phenotype associated with the variant.
(3) They are unaffected and have a variant incorrectly labeled pathogenic; they do not have susceptibility.

Determining that an individual is in state 1 is trivial. For the individuals who are apparently clinically unaffected, we set out to determine the likelihood of state 2 versus state 3.

We used *BRCA1*-related (MIM: 604370) or *BRCA2*-related cancer predisposition (MIM: 612555), which has a population prevalence of ∼1 in 400, or ∼0.25%, as our example disorder and incorporated clinical and genotypic data from participants with variants in these genes into our estimates.^18^ We estimated that 50% of individuals with this disorder harbor a likely pathogenic (LP) variant in one of those genes and that the likelihood of finding an LP variant in a person who does not have *BRCA1-* or *BRCA2*-related cancer predisposition is about 1 in 1,000.^18^ A justification of these estimates and the pedigree of the example family we describe below are presented in the supplemental methods.

Using these estimates, we calculated the baseline posterior probability that a randomly selected individual from the population with an LP variant (i.e., a secondary-findings recipient) is affected with a CMD of *BRCA1-* or *BRCA2*-related cancer predisposition. Note that this first calculation was ignorant of the CMD status of the secondary finding testee. As shown in Table 1, the baseline probability of a positive CMD of *BRCA2*-related cancer predisposition was 58.2% until clinical and genotypic data from that individual and their family were loaded into risk estimates.

**Table 1:** Initial probability of CMD

	**Probability affected (CMD+)**	**Probability unaffected (CMD−)**
Prior probability	0.0025 (A)	0.9975 (B)
Conditional probability of an LP variant	0.5 (C)	0.0009 (D)
Joint probability	0.00125 (E)	0.000898 (F)
Posterior probability	58.2% (G)	41.8% (H)

(A) and (B) are the prior probabilities derived from the population prevalence of *BRCA1- or BRCA2*-related cancer predisposition disorder. (C) is the conditional probability of identifying a likely pathogenic (LP) variant in a person with the disorder. (D) is the probability of identifying an LP variant in a person who is known to not have the disorder. (E) is the product of (A) and (C). (F) is the product of (B) and (D). Posterior probabilities in the last row of the table are derived per standard Bayesian arithmetic: (G) = (E)/[(E) + (F)] and (H) = [(F)/[(E) + (F)]. Decimals are used for most probabilities, but percentages are used for the posteriors for clarity.

The family’s clinical and genotype data can now be considered. Our calculations here are based on a family with an LP *BRCA2* variant and several individuals with cancer (family ID 8334; see supplemental methods for pedigree). We used the epidemiologic meta-analyses provided by the ASK2ME (All Syndromes Known to Man Evaluator) team (https://ask2me.org/) with the age set to the minimum value and no cancers selected to derive the lifetime risk data. The proband, her mother, and maternal grandmother were all positive for the same LP variant in *BRCA2*, the latter two via the cascade testing we provided. For each of these individuals, the likelihood of cancer was estimated from the ASK2ME data, under the scenario that they were affected versus unaffected with *BRCA2*-related cancer predisposition. The joint probabilities of health statuses observed in the genotype-positive individuals in the family, assuming the family was affected (CMD+) or unaffected (CMD−) with *BRCA2*-related cancer predisposition, can then be calculated (Table 2).

**Table 2:** Conditional probabilities of observed health status for specific genotyped individuals in family 8334

**Clinical and genetic data of each individual**	**Probability affected (CMD+)**	**Probability unaffected (CMD−)**
38-year-old female without cancer (proband)	0.926 (J)	0.995 (K)
Female diagnosed with breast cancer at age 47 years (mother)	0.188 (L)	0.0171 (M)
Female diagnosed with breast cancer at age 55 years (maternal grandmother)	0.307 (N)	0.0367 (P)
Joint probability	0.053 (Q)	0.00062 (R)

Clinical data observed for three individuals from family 8334 are provided in each row; each of these individuals has the same likely pathogenic variant in *BRCA2*. (J), (L), and (N) are likelihoods of observing that individual’s health status if the family had a clinicomolecular diagnosis (CMD) of *BRCA2*-related cancer predisposition, while (K), (M), and (P) are the same likelihoods if the family does not have this CMD. (Q) is the product of (J), (L), and (N). (R) is the product of (K), (M), and (P).

We then used the posterior probabilities from Table 1 as the prior probability for the next calculation, which now includes the combined (multiplied) cascade testing family data from Table 2. Taking genotypic and phenotypic family data into account raised the likelihood of a positive CMD for *BRCA2*-related cancer predisposition in this example family to 99.2% based on the presence of early-onset breast cancer in two members of this family who harbor the variant (Table 3). This fits with clinical intuition, as such a family is more likely to have *BRCA2*-related cancer predisposition rather than having coincidental breast cancer unassociated with those disorders. That the proband was not then currently affected with cancer does not much detract from this conclusion, which again fits with clinical intuition.

**Table 3:** Bayesian probability of CMD for family 8334

	**Probability affected (CMD+)**	**Probability unaffected (CMD−)**
Prior probability	0.582 (S)	0.418 (T)
Conditional probability	0.053 (U)	0.00062 (V)
Joint probability	0.031 (W)	0.00026 (X)
Posterior probability	99.2%	0.8%

Shown is the Bayesian calculation of probabilities that family 8334 is affected (99.2%, CMD+) and unaffected (0.8%, CMD−) by *BRCA2*-related cancer predisposition when phenotype data from genotyped members of the family were incorporated. (S) and (T) were derived from the posterior probabilities (G) and (H), respectively, shown in Table S1. (U) is the value of (Q) from Table S2, and (V) is the value of (R) from Table S2. (W) is the product of (S) and (U), and (X) is the product of (T) and (V). Posterior probabilities in the last line of the table were derived per standard Bayesian arithmetic. Decimals are used for most probabilities, but percentages are used for the posteriors for clarity.

We next considered inclusion of relatives who had not been tested for the variant but were related to an individual who is positive for the variant. These calculations are shown in the supplemental methods. For our example family, there were only two relevant untested individuals, both without cancer, and incorporating these data caused the resultant posterior probability of disease to decline modestly to 98.9%. Again, this matches clinical intuition because the presence of a few family members without cancer does not much reduce the likelihood that that disorder is present.

We assumed that the three variants that have been associated with this susceptibility in the Ashkenazi Jewish population had a probability of pathogenicity of 100% and excluded participants with these variants as well as participants with more than one cancer-associated variant in the family or phenotypes absent in the ASK2ME database in this analysis.

## Results

### Participant demographics

**Figure 1:**
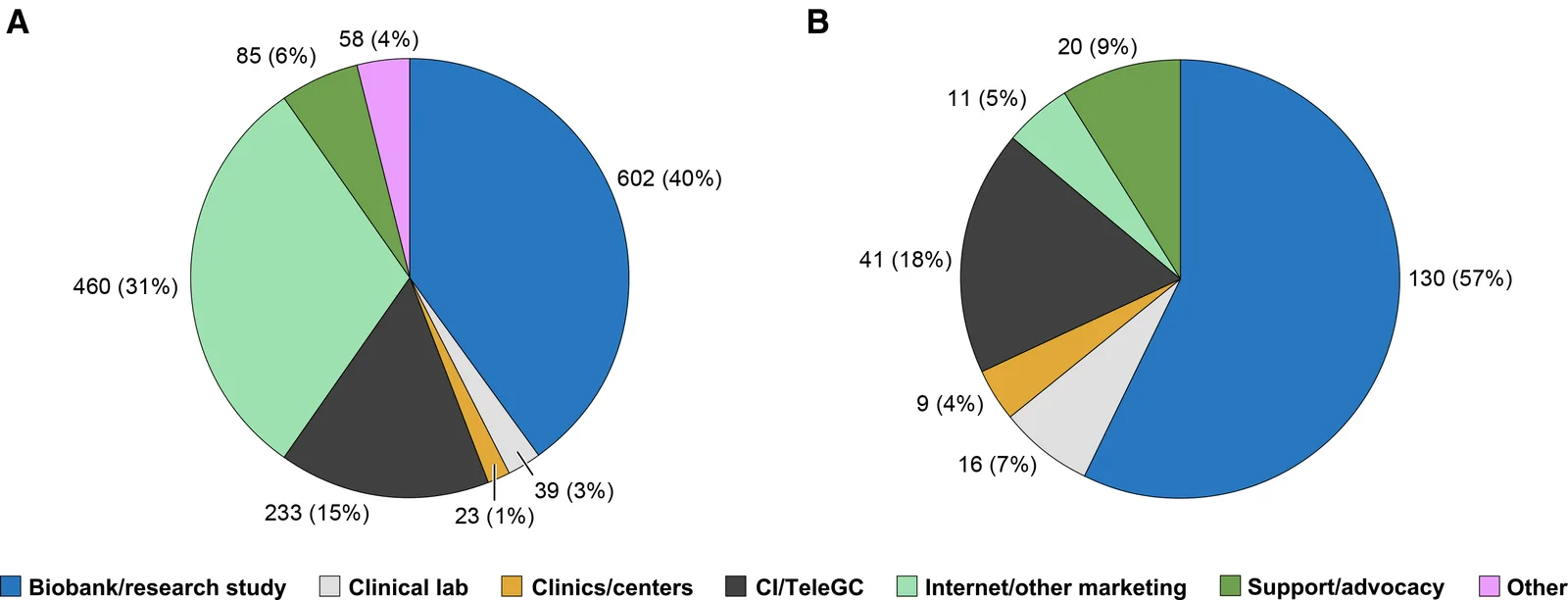
Study referrals and enrollments (A) Referrals of potential participants in this study by source; total = 1,500. (B) Enrollment of participants in the study by source; total = 227. DTC, direct-to-consumer testing company; TeleGC, a telehealth genetic counseling service provider, typically under contract to a genetic testing laboratory.

We froze this analysis at the first 1,500 inquires and referrals, the last of which occurred on June 29, 2023, and report results through December 31, 2023. The largest number of referrals were from biobanks and other research programs (40%), followed by our internet marketing efforts (31%) and consumer-initiated testing labs and telephone genetic counseling services (15%; Figure 1A). The 227 secondary findings recipients we enrolled in this interval reflect our broad recruitment efforts and a variety of sequencing settings (Figure 1B). Many prospective participants (*n* = 172) were not able to provide us with CLIA-validated reports demonstrating the presence of a secondary finding. An additional 83 submitted CLIA-validated reports of variants classified as variants of uncertain significance (VUSs) by the source laboratory; none of these individuals were enrolled in this study.

Demographic data were collected on individuals who consented to the study. Participants self-identified as White (84%), Black or African American (6%), and mixed race or other races (about 5%). About 6% indicated that they were Hispanic or Latino, and 67% were female. The median self-reported annual family income was US$80–US$99,000, and the median educational attainment was a bachelor’s degree. Of the 227 screen-eligible individuals we enrolled, six were reclassified as screen failures after consent, three declined continued participation shortly after consent (one actively and two passively/lost to follow up), and 18 did not meet eligibility criteria for genotyping and phenotyping efforts as outlined above.

### Variant classification and study workflow

**Figure 2:**
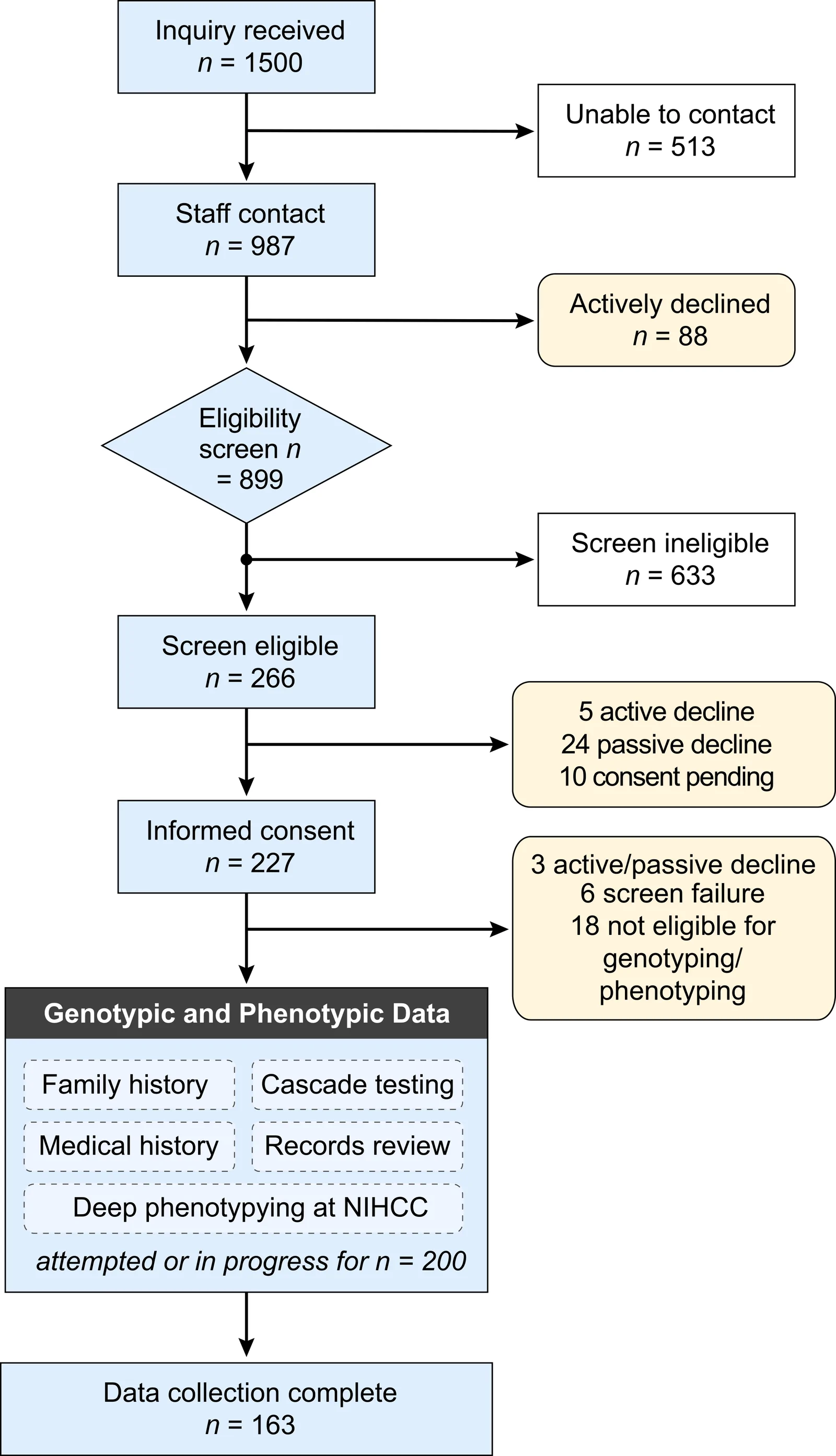
Study flow Shown is a flow diagram of the study organization and the flow of participants through the various steps of the study. Of the 1,500 inquiries, 266 (18%) screened eligible.

We collected as much genotypic, family history, and phenotypic data as practical from the remaining 200 active participants. Cascade testing was offered to the families of 76 participants (114 total living relatives) and performed in 35 families comprising 69 individuals, 34 of whom were positive for the secondary finding variant. We completed genotypic/phenotypic data collection efforts for 163 (see Figure 2 for a study flow diagram).

We re-classified the variants of 11 enrolled individuals as VUS or below when the source laboratory assessed them as LP or pathogenic. Of the 163 individuals for whom we completed data collection and report here, we also re-classified 16 variants as LP when these variants had been reported as pathogenic by the issuing laboratory (Table S2). This latter reclassification did not affect their eligibility but could affect our estimate of the likelihood of disease for those harboring *BRCA1* or *BRCA2* variants (see below).

Among 163 participants for whom data collection was complete, we identified 108 distinct variants in 25 genes (Table 4). Individuals with cancer susceptibility variants were the most common among disease groups, comprising 8 of the 25 genes and 89 affected families. Variants were present in 15 cardiovascular genes among 46 families. The two remaining genes were *RYR1* (10 families [MIM: 180901]) and *HFE* (18 families [MIM: 613609]). While we expected to encounter cancer genes most commonly (they comprise 28 of 78 genes on the ACMG 3.1 secondary findings list^19^), the distribution of *BRCA1* (*n* = 10, 17%) versus *BRCA2* variants *BRCA2* (*n* = 49, 83%) in our cohort was notable. This is markedly different from the distribution of the two genes in individuals who undergo indicated testing, which is 66% *BRCA1* and 34% *BRCA2*,^18^ whereas the 99% confidence interval of our sample is 7.8%–34.1% *BRCA1* and 65.9%–92.1% *BRCA2*.

**Table 4:** Number of individuals identified with a secondary finding by gene and number of unique variants

**Gene**	**Individuals**	**Unique variants**
**Cancer**		

*BRCA2* (MIM: 600185)	49	37
*BRCA1* (MIM: 113705)	10	5
*RET* (MIM: 164761)	12	8
*MSH6* (MIM: 600678)	7	5
*PMS2* (MIM: 600259)	7	7
*MLH1* (MIM: 120436)	1	1
*PALB2* (MIM: 610355)	3	3
*BMPR1A* (MIM: 601299)	1	1

**Cardiovascular**		

*MYBPC3* (MIM: 600958)	9	8
*LDLR* (MIM: 606945)	8	8
*MYH7* (MIM: 160760)	5	4
*SCN5A* (MIM: 600163)	4	4
*APOB* (MIM: 107730)	4	1
*TTN* (MIM: 188840)	3	3
*KCNQ1* (MIM: 607542)	3	3
*DSC2* (MIM: 125645)	2	2
*DSG2* (MIM: 125671)	2	2
*DSP* (MIM: 125647)	1	1
*KCNH2* (MIM: 607542)	1	1
*FBN1* (MIM: 134797)	1	1
*LMNA* (MIM: 150330)	1	1
*PKP2* (MIM: 602861)	1	1
*COL3A1* (MIM: 120180)	1	1

**Other**		

*HFE* (MIM: 613609)	18	1
*RYR1* (MIM:180901)	10	4
Totals	163	108

### Defining secondary findings for genomics implementation research

Green et al. defined secondary findings in the clinical diagnostic setting as “results that are not related to the indication for ordering the sequencing but that may nonetheless be of medical value or utility to the ordering physician and the patient.”^1^ Recognizing the extent to which this definition has been adapted for opportunistic screening in other sequencing settings, we initially developed a working definition of a secondary finding for the purposes of this study that evaluated three successive criteria:

(1) a reasonable clinician would judge the testing to not be indicated (secondary),
(2) the reporting laboratory understood the finding to be unrelated to the testing indication, and
(3) the participant perceived the finding as unexpected and/or unrelated to the indication for testing.

We typically performed these in order, and if a criterion pointed to the result being primary, we determined them ineligible and did not evaluate the subsequent criteria. After screening the first 1,000 prospective participants for eligibility, it became apparent that criterion 1 had to be dropped because of the substantial number of cases where we could not apply it reliably. In fact, when we applied NCCN guidelines to 59 pedigrees of participants with *BRCA1* or *BRCA2* variants, over half (51%, *n* = 30) met criteria for diagnostic testing for a hereditary cancer syndrome. A diagnosis of breast cancer under age 50 years was present in two probands, four first-degree relatives, and 10 second-degree relatives (see Table S3 for additional details). In many cases, it appeared that clinicians either poorly addressed this issue and/or that their original understanding and intent could not reliably be determined by us. As well, for participants sequenced as part of research studies or engaging in consumer initiated genetic testing, the role of ordering clinician did not apply. We give examples of eligibility determinations in Table 5, and how we evaluated these criteria over the course of the study is further considered in the discussion.

**Table 5:** Case examples demonstrating challenges in defining the secondary nature of genomic findings

**Case example**		**Discussion**
1.	40-year-old woman orders DTC ancestry genetic testing and learned of pathogenic *APOB* variant. Proband reported 15-year history of high cholesterol, and family history was significant for high cholesterol with pediatric onset in her brother and two children and significant paternal family history of coronary artery disease resulting in death from cardiac events.	Secondary. Early-onset hypercholesterolemia and/or heart disease in family members coupled with this participant’s personal history of hypercholesterolemia are strongly suggestive of familial hypercholesterolemia (FH), which is underrecognized clinically (e.g., Zimmerman et al.^20^). This participant described her result as very surprising and reported that none of her clinicians had ever mentioned a heritable component to high cholesterol to her; she was enrolled in the study.
2.	62-year-old participant in a biobank learns of pathogenic *BRCA2* variant, which she describes as very surprising. Before learning her result, she reported feeling reassured that her primary care physician advised against genetic testing for breast cancer susceptibility despite her family history of breast cancer in her mother and maternal aunt in their 50s.	Secondary. Diagnostic testing for hereditary breast and ovarian cancer could have been indicated in this case, and this participant actively sought clinical testing. Unlike other biobank participants we did not enroll, this participant did not join the biobank with the intention to understand risks for a known or suspected disorder, and she was enrolled in the study.
3.	A woman in her early 60s being treated for a paraganglioma underwent cascade testing for a pathogenic *SDHB* variant found in her daughter. Her daughter’s variant was found during clinical testing initiated by her daughter’s genetic counselor.	Primary. While this case illustrates a missed opportunity for diagnostic testing in an affected individual, this person was not enrolled in our study because her daughter urged her to have cascade testing for a known variant, and thus the finding was not unexpected, even as the proband reported being surprised to learn that it was possible to have genetic testing for her type of cancer.
4.	A man in his 40s seeks testing for a personal history of colon cancer. A maternal uncle had colon cancer at age 55 and his mother had endometrial cancer at age 49. A cancer panel of 100+ cancer-related genes found a pathogenic *MSH2* variant and a likely pathogenic *PALB2* variant. “Personal and family history of Lynch-syndrome-associated” cancers was listed as the indication for testing, and the counseling letter described the *PALB2* variant as “unexpected and unrelated to [your] Lynch syndrome.”	Only the *PALB2* variant is secondary. In addition to expressing surprise, the participant provided documentation that ordering provider considered this to be a secondary finding and citing lack of evidence connecting *PALB2* variants with colon and uterine cancers.

### Clinical utility of BRCA1 and BRCA2 secondary findings

We next set out to analyze the diagnostic positive predictive value of a secondary finding, similarly to how we conceptualized this in 2020^13^ using the concept of CMD, which describes an individual who both has that pathophysiologic state and harbors a gene variant that causes that state. Because many pathogenic and LP variants do not have a 100% probability of pathogenicity, ascertaining them from the general population should enrich for variants that are thought to be pathogenic or LP but are instead actually benign (as compared to ascertaining them in a high-risk diagnostic testing population).

We piloted this in the *BRCA1-* and *BRCA2-*related cancer predisposition genes because these comprised our largest single category of disease (*n* = 59 families) for which there are robust estimates of penetrance. Thirteen families were excluded from the Bayesian calculations but retained for considerations of clinical utility because they had one of the three variants in the Ashkenazi Jewish population. Another two families were excluded from all further considerations because in one the family was segregating both a *BRCA1* and *PALB2* variant and in another because two members of the family were affected by melanoma at an early age, but the epidemiologic data do not provide age-specific penetrance values that can support these calculations. We used the series of Bayesian calculations described under subjects and methods to determine the likelihood of a positive CMD of *BRCA1*- or *BRCA2*-related cancer predisposition for the remaining 44 families.

**Figure 3:**
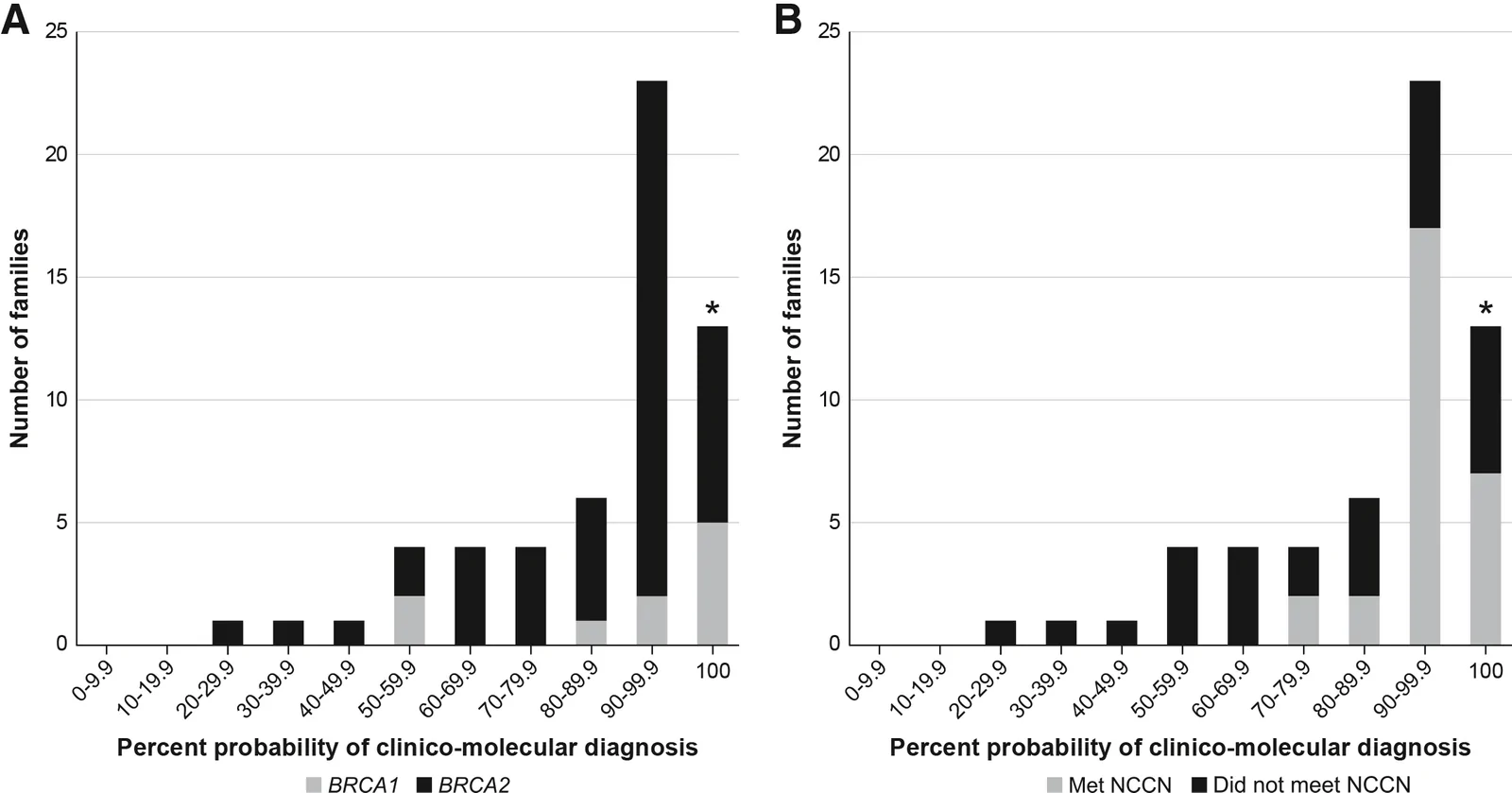
Posterior probabilities of CMDs (A) Distribution of posterior probabilities of clinicomolecular diagnoses (CMDs) of hereditary breast and ovarian cancer predisposition in 57 families with *BRCA1* (gray shading) or *BRCA2* variants (black shading). (B) Distribution of posterior probabilities of CMDs of hereditary breast and ovarian cancer predisposition in 57 families with *BRCA1* or *BRCA2* variants who met (gray shading) or did not meet (black shading) National Comprehensive Cancer Network (NCCN) criteria for clinical diagnostic testing. Families with Ashkenazi-associated variants (*n* = 13, denoted with an asterisk) were assumed to have 100% probability of CMD.

The posterior probability of a CMD of *BRCA1*- or *BRCA2*-related cancer predisposition in these families ranged from 26.2% to >99.9% (Figure 3A). Some pedigrees showed no apparent evidence of the phenotype, whereas others were extensive. Including families with Ashkenazi Jewish variants, the average probability of CMD was 86.9% (*n* = 57 families). The mean CMD probability in families who met NCCN criteria was 95.9% (*n* = 28), and it was 78.2% in families who did not meet NCCN criteria (*n* = 29; Figure 3B).

## Discussion

We successfully recruited a cohort of individuals with secondary findings and deeply evaluated and phenotyped them for that finding, including cascade testing of potentially affected relatives. The starting point of our work was that we did not assume that because an individual harbored a pathogenic or LP variant that they necessarily had the associated monogenic disease entity. Instead, we framed this as a probabilistic determination for which we developed a method to measure this probability This approach allowed us to pilot a method of incorporating familial genotypic and phenotypic data to refine risk estimates for individuals with *BRCA1* and *BRCA2* variants. We also operationalized a definition of secondary findings for genomic implementation research that anticipates population-level screening and recognizes the varied contexts in which opportunistic genomic screening is currently performed.

Three-fourths of eligible participants received their finding through participation in a biobank or a research study (*n* = 130, 57%) or through consumer-initiated genetic testing (*n* = 41, 18%, 75% in sum). It is concerning that less than one-quarter of our participants are derived from the target population of recommendations for secondary findings return.^1^ This could be for several possible reasons. The first is that there may have been a decline in the receipt of secondary findings. However, anecdotal reports from several large clinical laboratories suggest that uptake ranges from 80% to 90% (unpublished data, L.G.B.). The second is that despite our broad recruitment efforts, biobank and consumer-initiated testees were more likely to enroll in a secondary finding study.

Initially, we endeavored to exclude individuals whose genetic testing result was actually primary in that the identified variant related to the clinical indication for the testing. It was challenging to make these determinations because the original ACMG definition of secondary findings can only be precisely applied in the clinical diagnostic setting, and we did not have access to records from the ordering clinician that might support this determination. We had to instead rely on the laboratory report (which in some cases made clear it was primary) or the history described by the participant. We came to recognize that this challenge is difficult to resolve and that our study included some participants whose result would be considered by some to be primary instead of secondary. Indeed, over half of the families with *BRCA1* or *BRCA2* secondary findings we ascertained here met NCCN criteria for clinical testing in our retrospective review. Our observation of these occurrences is consistent with studies demonstrating that genetic testing is substantially underutilized in clinical practice and that these serious disease risks are not being appropriately addressed.^21^^,^^22^^,^^23^^,^^24^^,^^25^^,^^26^ While secondary findings evaluation is inadequate, alone, to address these health care systems genomic testing deficiencies, it is important to highlight these failures. Opportunistic screening for any disorder should not function as a substitute for diagnostic testing in scenarios where diagnostic testing is indicated. It is concerning that this seemed to be the case for many of our participants. Negative results are not followed up in the secondary findings paradigm, and high VUSs (which can be used for patient management decisions in diagnostic testing) are not returned. These are important considerations for future population screening and opportunistic genomic screening should encompass these scenarios until there is a successful approach to increasing the uptake of diagnostic genetic testing. Finding some of these cases is better than finding none.

The single largest category of secondary findings in our study was for hereditary breast and ovarian cancer predisposition, which reflects the distribution of variants reported in large sequencing studies.^3^^,^^27^^,^^28^^,^^29^^,^^30^ We noted the much lower proportion of *BRCA1* to *BRCA2* secondary findings (10:49 or 17% and 83%), which was highly statistically significantly different from the expected numbers from a diagnostic population (expected proportion ∼39:20 or 66% and 34%).^18^ This skewing is more extreme than, but similar to, what was observed in the data reported from the Geisinger MyCode study,^28^ which included 95 (35.6%) *BRCA1* variants and 172 (64.4%) *BRCA2* variants. Our data are significantly more skewed toward *BRCA2* than the MyCode paper (χ^2^ = 0.0.0083). This may be because we excluded some diagnostic (primary) cases, whereas the MyCode paper looked at everyone who enrolled in MyCode, irrespective of whether they were primary or secondary findings. Our data strengthen the conclusion of Manickam et al. that reduced penetrance of *BRCA2*-related cancer predisposition compared to *BRCA1* related would shift the ascertainment toward *BRCA2* in a secondary findings context.^28^

We recognized in this cohort a wide range of family test result scenarios and that this range of outcomes may be challenging for some clinicians to interpret and correctly assess risks to make prudent management recommendations. Families at one end of this spectrum had multiple affected family members and high rates of cascade testing (e.g., a family with five individuals affected by hypercholesterolemia who all shared a pathogenic *APOB* [MIM: 107730] variant). Others had less striking phenotypic attributes and/or cascade data were sparse or absent, and it was clear that we needed a quantitative metric to assess risks. Clinical intuition is probably sufficient when the family history is extensive, when cascade testing is performed and is informative, and there are numerous affected individuals; it is obvious that such families have a high or near certain likelihood of a positive CMD and that preventive management should be pursued. However, this is not always the case, and in such cases, a quantitative assessment of the likelihood of a CMD is essential for prudent management decisions.

The crucial recognition for managing families with secondary findings is that not all variants that are returned in this testing context will turn out to be causative of the associated disease. This is important in genomic opportunistic screening because of the selection bias of that testing setting, which is skewed toward less affected families. This bias enriches ascertainment for variants that are thought to be pathogenic or LP but are, in fact, benign. This selection bias can be framed in terms of Bayesian probability. In secondary findings, the prior probability of disease is approximately equal to the population incidence of the disorder (which ranges from 1 in 400 to 1 in 50,000 for the ACMG gene list), in contrast to diagnostic testing, where the prior probability of disease is commonly 1 in 2 or higher. While a pathogenic or even LP variant is typically sufficient for a valid CMD in diagnostic testing (because of the high prior probability of disease), it may not be sufficient in secondary findings testing (where the prior probability is low).^31^ Therefore, clinicians need tools to assess these risks to refine management recommendations for secondary findings recipients.

We developed a Bayesian approach to this assessment using our largest subset of results, the *BRCA1*- and *BRCA2*-related cancer predisposition disorders. This approach leverages the well-established approach in Mendelian genetics of cascade testing of the relatives of a proband. We show a wide range of results from 26.2% to 99.9% likelihood of CMD (excluding the Ashkenazi-associated variants that we assumed to be of certain pathogenicity). The average probability of a diagnosis was 86.9%, but, as expected, the distribution was not normal. These calculations account for the wide range of available data for a given family, quantifying the clinical aphorism that “the absence of evidence is not evidence of absence.” Taking the extremes of our distribution of CMD likelihood, we suggest that an individual harboring a *BRCA1* or *BRCA2* variant with a 26.2% probability of actually having that disorder should be managed very differently than one with a 99.9% probability. While individual preferences and tolerance of uncertainty are paramount, prudent management might skew toward careful monitoring (e.g., regular breast MRI and ovarian cancer screening) rather than prophylactic surgery when the CMD likelihood is low. An extremely undesirable outcome of genomic screening would be unnecessary prophylactic surgery for a variant with a low probability of CMD. The careful monitoring scenario also allows for evidence to evolve over time. The family history may change, unaffected individuals may become affected, potentially informative family members resistant to testing may change their minds, and improved data on, or methods for, variant classification may become available. In such situations, the risk can be recalculated and the management strategy reassessed.

It is worth noting that we are assessing yield of secondary findings but have not attempted to assess the penetrance of disorders identified through opportunistic screening. When an individual or family with a secondary finding is identified but no signs of the disorder are evident, there are several possibilities: (1) the variant is actually benign, and the patient/family does not have the disorder; (2) the patient/family has the susceptibility disorder but has no manifestations of disease; and (3) the patient has the susceptibility disorder but has manifestations of the disease that are not clinically evident or detected. Only the second possibility is penetrance. Several recent publications have concluded from population ascertainment study designs that penetrance is lower than expected.^30^ In fact, these claimed observations of reduced penetrance reflect all three of the factors enumerated above and are not, per se, measurements of penetrance.

### Limitations

Despite our attempts to ascertain secondary findings recipients from the clinical diagnostic setting, most of our participants learned their results outside of this context, requiring us to adapt the ACMG definition; further refinement of this definition may be needed. As well, while we argue that our results from mostly non-clinical testing is generalizable to clinical testing, there may be limitations to that generalizability. The distribution of secondary findings reported by our participants may differ from the distribution of variants in clinical testing settings. Ascertainment biases in our study may cause our data to not reflect the broader population of secondary findings recipients. We relied heavily on participants’ reports of the testing context and personal and family histories, all of which are subject to recall bias. We offered free cascade testing to relatives and invested significant time to interview relatives and gather and review outside records to generate the data needed for the Bayesian analyses we piloted here.

### Conclusions

The key conclusion from this work is that, in a secondary findings context, the probability that the patient has the implicated monogenic disease entity is not equal to the probability of pathogenicity of the variant. A *BRCA1* variant at the lower bound of LP has a probability of pathogenicity of 90%. A secondary findings recipient with such a variant has a probability of actually having *BRCA1*-related cancer predisposition that could be higher or potentially much lower than 90%. This phenomenon is universal in medical testing – the testing context determines the prior probability of disease, and when that is coupled to a test result, it determines the posterior probability of disease. The clinician must take these diagnostic probabilities into consideration when managing patients. Individuals with a low probability of a CMD may be more appropriately managed with surveillance rather than prophylactic surgery. Such decisions are always dependent on shared decision-making between the provider and the patient, but good shared decision-making cannot happen if the CMD probability is significantly over- or underestimated. As well, these data have broad implications for policy-making. *BRCA1*- and *BRCA2*-related cancer predisposition are two of the more common monogenic disease entities in the ACMG Secondary Findings recommendations. For some of the rarer disorders, the CMD probabilities will, on average, be much lower than the estimates we show here. The secondary findings policy committee should consider whether the reporting thresholds for the rarer disorders should be raised to reduce the number of false positives. Finally, all of these estimates and decisions require robust epidemiologic data. Further studies of secondary findings and research support to develop robust epidemiologic meta-analyses will be crucial to support these critical medical decisions. As we move into broader modes of population screening, these issues will only become more salient.

## Data and code availability

There are restrictions to the availability of the dataset to preserve confidentiality of the participants. Certain de-identified data may be shared with qualified investigators upon request and in accordance with NIH data sharing policies.

## Acknowledgments

We acknowledge the generosity of the participants and their families who participated in our study. We are grateful to Kade McCulloch, Elisa Kucevic, Chalé Jacks, and Sara Rubovits for their assistance with data cleaning and the NIAID centralized sequencing team and the staff at Color Genomics for their efforts to refer participants to our protocol. This research was supported by the Intramural Research Program of the 10.13039/100000051National Human Genome Research Institute and 10.13039/100000060National Institute of Allergy and Infectious Diseases, National Institutes of Health. The contributions of the NIH author(s) were made as part of their official duties as NIH federal employees, are in compliance with agency policy requirements, and are considered Works of the United States Government. However, the findings and conclusions presented in this paper are those of the author(s) and do not necessarily reflect the views of the NIH or the US Department of Health and Human Services.

## Declaration of interests

J.C.S., E.W.M., A. Davidson, C.L.E., A. Darling, A.J.d.M., B.R., J.J.J., and L.G.B. were supported by intramural NIH grants 1ZIHG200387-12 and 1ZIHG200359-17. C.T. and C.M.W. were supported by intramural NIH grant 1ZICHG200418-04. K.L.L. and M.S. were supported by intramural NIH grant 1ZICAI001244-06. L.G.B. receives research support from Merck, Inc.; has previously served as a member of an Illumina advisory committee; and receives royalties from Wolters-Kluwer for his contributions to Up-To-Date.

## Supplemental information

Document S1. Tables S1 and S3 and Supplemental Methods

Table S2. Variant classifications

Document S2. Article plus supplemental information
