# Supplementary material for: Measuring disease likelihood in genomic ascertainment
Source: Am J Hum Genet. 2026 Apr 7;113(5):978–89. doi: 10.1016/j.ajhg.2026.03.009 (PMC13277679; doi:10.1016/j.ajhg.2026.03.009)
Supplement: Document S1. Tables S1 and S3 and Supplemental Methods [file mmc1.pdf]

The American Journal of Human Genetics, Volume *113*

Supplemental information

Measuring disease likelihood

in genomic ascertainment

Julie C. Sapp, Katie L. Lewis, Emily W. Modlin, Alana Davidson, Charlotte Linton Early, Adam H. Buchanan, Alexis Darling, Jacquelyn Mahder, Cara Z. McCormick, Allison J. de Moya, Brooke Rosenblum, Morgan Similuk, Kelly Tangney, Meghan C. Towne, Clesson Turner, Caralynn M. Wilczewski, Jennifer J. Johnston, and Leslie G. Biesecker

Table S1: List of Recruitment Modalities Employed for GSRP Pilot Phase (1500 Referrals) by Category

At least one inquiry or referral was received from each of the following sources during the pilot phase of GSRP:

Recruitment Mechanism	
**Invited Contact from Interested Individuals**	
Recruitment Modality Groupings, Details	Specific Source/Partner
Clinical Lab – included information about our study in reports with SF inviting recipients to contact us	Ambry<br>Fulgent<br>GeneDx<br>Invitae
Support/Advocacy Group – advertised or posted information about our study and invited membership to contact us	AMEN Support<br>Cure 4 Kids Foundation<br>EDS Society<br>FORCE<br>GlobalGenes<br>Marfan Foundation<br>PTEN Foundation<br>SADS (Sudden Arrythmia Death Society)<br>Smart Patients<br>TSC Alliance<br>VEDS<br>Other support/advocacy
Consumer-Initiated Testing/Tele-Genetic Counseling Company – advertised our study in newsletters and study announcements to clients and/or referred patients to us	Color<br>InformedDNA<br>PWN
Internet/Other Direct Marketing – directly invited interested individuals to contact us	Clinical Trials.gov<br>Email Newsletter<br>Facebook<br>Google<br>Instagram<br>LinkedIn<br>NIH Website<br>Paid digital marketing
**Direct Referrals of Patients/Participants**	
Recruitment Modality Groupings, Details	Specific Source/Partner
Clinics/Centers – directly referred patients to our study after gaining patients' permission to do so	Advent Health<br>Albany Medical Center<br>Children's Hospital of Philadelphia<br>Children's Hospital of Pittsburgh<br>Duke - GC<br>GC at Mount Carmel<br>Georgetown<br>Johns Hopkins University<br>Seattle Childrens<br>Spectrum Health<br>Stanford<br>Texas Childrens<br>University of Utah Huntsman Cancer<br>University of Vermont
Biobank/Research Study – directly referred participants to our study after gaining participants' permission to do so	ClinSeq<br>Eye Institute, Clinical Study<br>HealthyOregon<br>MyCode/Geisinger<br>NIAID<br>SGFS<br>UDN at Stanford<br>Wisdom Study

Table S3. Narrative descriptions of participants meeting NCCN criteria

Participant ID	Attributes meeting NCCN criteria
1018	Proband had breast cancer at age 36 and is of Ashkenazi Jewish Ancestry
1030	Paternal grandmother died of breast cancer in her 30's
1056	Proband's daughter had breast cancer at 35, proband's father had pancreatic cancer in his 80's
1203	Proband's maternal aunt had breast cancer in her 20's
1362	Proband's materal grandmother died of ovarian cancer at age 70
1729	Proband's maternal grandmother died of breast cancer at age 45
1778	Proband's mother had pancreatic cancer in 90's, and proband is of Ashkenazi Jewish ancestry
1925	Proband's paternal aunt had ovarian cancer at unknown age
1936	Proband had breast cancer in her 30's
2139	Proband's maternal grandmother died of breast cancer at age 45 and proband is of Ashkenazi Jewish ancestry
2404	Proband had prostate cancer at 62, proband's sister had breast cancer at age 50 and proband's paternal grandmother had breast cancer in her 60's
2485	Proband's maternal grandmother died of breast cancer at age 45.
2644	Proband's paternal aunt diagnosed with breast cancer in her 40's
3155	Proband's maternal grandfather died of metastatic prostate cancer at age 50
3579	Proband's paternal grandmother diagnosed with breast and ovarian cancer in her 70's
4382	Proband's paternal aunt had breast cancer in her 60's and pancreatic cancer at age 79 and one of this individual's daughters (paternal first cousin to proband) was diagnosed with breast cancer at age 55 and died of breast cancer at age 59
4408	Proband's father died of pancreatic cancer at age 42, proband's paternal aunt died of breast cancer at age 55, and proband's paternal first cousin died of breast cancer in her late 50's
4887	Proband's paternal grandfather died of pancreatic cancer at age 72 and proband is of Ashkenazi Jewish ancestry
5450	Proband's maternal grandmother was diagnosed with ovarian cancer at age 65 and died at age 68
5889	Proband's maternal aunt diagnosed with breast cancer and died at age 48 after recurrence.
5944	Proband's mother had breast cancer diagnosed in her 50's and proband is of Ashkenazi Jewish Ancestry
6209	Proband's paternal aunt had ovarian cancer at age 70
6703	Proband's paternal grandmother died of breast cancer at age 43 and proband is of Ashkenazi Jewish Ancestry
7196	Proband's mother diagnosed with ovarian cancer in her early 40's and proband's materal grandmother died of ovarian cancer at age 54
7704	Proband's paternal aunt died of breast cancer in her 40's and proband's paternal half sister died of breast cancer at age 38
7907	Proband's mother had breast cancer diagnosed in her 40's
8305	Proband's paternal grandmother was diagnosed with breast cancer in her 40's and proband's paternal ancestry is Ashkenazi Jewish
8334	Proband's mother diagnosed with breast cancer at age 47, proband's maternal grandmother had breast cancer in her 50's
8960	Proband's paternal grandmother diagnosed with breast cancer at age 33 and died at age 47 following several recurrences
8934	Proband's maternal grandmother died of breast cancer before age 30

Supplemental Methods

Bayesian Calculations for the Probability of Disease Given a Variant and Family History

Here we define all secondary finding disorders (diseases) as susceptibilities with a specific molecular etiology (e.g., clinico-molecular diagnosis (CMD) of *MLH1*-related Lynch syndrome; see Katz et al for a definition of CMD[1]). Variants have a probability of pathogenicity, and most are less than 100%. A small number of variants have, for practical purposes, a pathogenicity of 100%. If an individual harbors a variant that has a pathogenicity of 100% (certain to be pathogenic (P)) then that individual is, by definition, affected by that susceptibility disorder (even if they are not penetrant). If the variant is of <100% pathogenicity, then the individual may be in one of three states:

1) They are affected and penetrant
2) They have a pathogenic variant but do not have manifestations of the disorder - this individual has a positive CMD and is non-penetrant
3) They have a variant that is thought to be pathogenic, but is not - this individual does not have the susceptibility and has a negative CMD

We set aside state 1 as straightforward because it is overwhelmingly likely, nearly certain, that such an individual has a CMD of the susceptibility disorder. Individuals in both states 2 and 3 appear clinically unaffected but are critically distinct. The example we consider here is a likely pathogenic (LP) variant in *BRCA2*. This variant has a probability of pathogenicity of 90-99%. The example disease we consider is *BRCA1*- or *BRCA2*-related cancer predisposition which has a population prevalence of ~1/400, or ~0.25%[2]. We estimate that 75% of individuals who have a CMD of *BRCA1*- or *BRCA2*-related cancer predisposition will be found to harbor a P or LP variant.

In the general population, some individuals will harbor a variant that is classified as P or LP, but is actually benign, and those individuals have a negative CMD (state 3 above). We estimate that the likelihood of finding a P/LP variant in a person without a CMD of *BRCA1*- or *BRCA2*-related cancer predisposition is about 1/1,000 ref[3]. Some justification of this estimate is considered here.

Estimates of population prevalence of P/LP variants:
gnomAD v2.1.1, downloaded October, 2023
BRCA1 179 P/LP variants in 129,200 alleles = 64,600 people = .00277 = 1/360
BRCA2 216 P/LP variants in 129,156 alleles = 64,578 people = .00334 = 1/298
Total prevalence of P/LP variants is 0.00611 = 1/163
This is ~2.5X the estimated population prevalence of *BRCA1*- or *BRCA2*-related cancer predisposition. Taken at face value, this would suggest that three to four randomly selected individuals per thousand harbor a variant classified as P/LP that is in fact benign (this would not be true for individuals selected for a positive family history of disease). Therefore, an estimate of a likelihood of a P or LP variant in a person who does not have *BRCA1*- or *BRCA2*-related cancer predisposition (state 3 above) of one per thousand (0.001) is reasonable. We then separated P and LP variants, estimating that 90% of the false positives would be LP variants and 10% would

be P. An estimate of substantially more or less than that is implausible (but alternate assumptions are tested below, see Supplemental Methods Table 6).

We separated P/LP variants for the diagnostic yield numbers. We estimated that about 2/3 of the variants in individuals with a CMD of *BRCA1*- or *BRCA2*-related cancer predisposition were LP and 1/3 P. Therefore, we partitioned the 0.75 probability for finding a P/LP variant in a CMD-positive person to be 0.5 for LP and 0.25 for P.

The next step was to calculate the posterior probability of CMD based on the pathogenicity of the secondary finding variant given these estimates. Note that this first calculation was ignorant of the CMD status of the secondary finding testee. The table below is identical to Table 1 in the main text and describes the Bayesian probability of CMD of *BRCA2*-related cancer predisposition in a secondary finding recipient with a LP *BRCA2* variant.

Supplemental Methods Table 1: Initial Probability of Clinico-Molecular Diagnosis (CMD)[a], Not Considering Affection Status of Family Members

	Probability affected (CMD+)	Probability unaffected (CMD-)
Prior probability	.0025 (A)	.9975 (B)
Conditional probability of a LP variant	.5 (C)	.0009 (D)
Joint probability	.00125 (E)	.000898 (F)
Posterior probability	58.2% (G)	41.8% (H)

[a] (A) and (B) are the prior probabilities derived from the population prevalence of *BRCA1- or BRCA2*-related cancer predisposition disorder. (C) is the conditional probability of identifying a likely pathogenic (LP) variant in a person with the disorder. (D) is the probability of identifying a LP variant in a person who is known to not have the disorder. (E) is the product of (A) and (C). (F) is the product of (B) and (D). Posterior probabilities in the last line of the table are derived per standard Bayesian arithmetic: (G) = (E)/[(E) + (F)] and (H) = [(F)/[(E) + (F)]. Decimals are used for most probabilities, but percentages are used for the posteriors for clarity.

Our conclusion was that the baseline probability of a positive CMD of *BRCA2*-related cancer predisposition in an individual selected randomly from the population (i.e., a secondary finding recipient) who is harboring a *BRCA2* variant classified as LP was 58.2% until clinical data are loaded into risk estimates.

The clinical and genotype data of the family were next considered to refine the CMD estimates. This is based on the fact that even though these families were ascertained opportunistically via secondary findings return, clinical data such as cascade testing results and the affection status of the secondary findings recipient and their family members alter the likelihood of a positive CMD and may substantially refine risk estimates.

We used an actual family for this calculation, family ID 8334 (pedigree shown below). Through our research program, we offered cascade testing to members of this family. The proband, her mother, and maternal grandmother were all positive for the same LP variant in *BRCA2*. Because the proband did not know the precise age at which her maternal grandmother was diagnosed with breast cancer, we estimated this individual's age of diagnosis at 55 years.

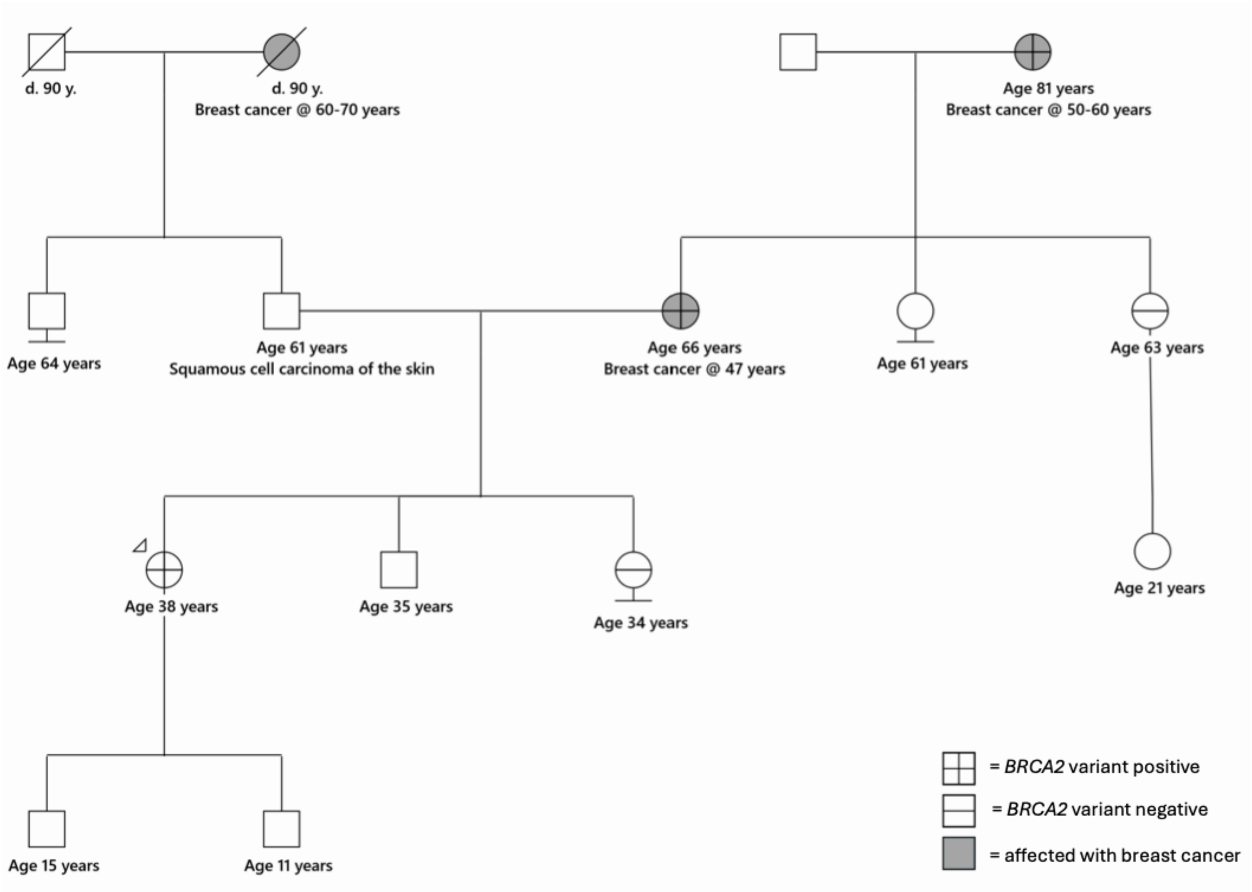

Supplemental Figure 1. Pedigree of a family identified through a secondary finding to harbor a likely pathogenic variant in *BRCA2*. This figure shows the phenotype and genotype status of the family members following cascade testing and clinical records gathering by our research team.

To make the calculations shown below in Supplemental Methods Table 2 (identical to Table 2 in the main text), we used the epidemiologic meta-analyses provided by the ASK2ME All Syndromes Known To Man Evaluator) team (https://ask2me.org/), with the age set to the minimum value and no cancers selected, to derive the lifetime risk data from their meta-analyses. These data provide age- and sex-specific risks for each cancer type. We then evaluated the pedigrees, and the cascade testing results for individuals who harbored the variant.

The likelihood of cancer for each individual with cancer in family 8334 was estimated from the ASK2ME data, under the alternative scenarios that they were affected versus unaffected with *BRCA2*-related cancer predisposition.

Supplemental Methods Table 2: Conditional Probabilities of Observed Health Status for Specific Genotyped Individuals in Family 8334[a]

Clinical and genetic data of each individual	Probability affected (CMD+)	Probability unaffected (CMD-)
38-year-old female without cancer (proband)	0.926 (J)	0.995 (K)

Female diagnosed with breast cancer at age 47 years (mother)	0.188 (L)	0.0171 (M)
Female diagnosed with breast cancer at age 55 years (maternal grandmother)	0.307 (N)	0.0367 (P)
Joint probability	0.053 (Q)	0.00062 (R)

[a] Clinical data observed for three individuals from Family 8334 are provided in each row; each of these individuals has the same likely pathogenic variant in *BRCA2*. (J), (L), and (N) are likelihoods of observing that individual's health status if the family had a clinico-molecular diagnosis (CMD) of *BRCA2*-related cancer predisposition while (K), (M), and (P) are the same likelihoods if the family does not have this CMD. (Q) is the product of (J), (L), and (N). (R) is the product of (K), (M), and (P).

Note that J is lower than K, which is a conditional probability that lowers the probability they are CMD+, because it takes into account the proband, who does not have cancer. In contrast, note that L is greater than M and N is greater than P. These two conditional probabilities raise the probability of CMD because these women were affected by cancer. The posterior probabilities from Supplemental Methods Table 1 were used as the prior probabilities for the next Bayesian calculation, which now includes the combined (joint) cascade testing family data derived from Supplemental Methods Table 2 as shown in Supplemental Methods Table 3 (the same as Table 3 in the main text):

Supplemental Methods Table 3: Bayesian Probability of Clinico-Molecular Diagnosis (CMD) for Family 8334[a] Including Information from Genotyped Family Members

	Probability affected (CMD+)	Probability unaffected (CMD-)
Prior probability	0.582 (S)	0.418 (T)
Conditional probability	0.053 (U)	0.00062 (V)
Joint probability	0.031 (W)	0.00026 (X)
Posterior probability	99.2%	0.8%

[a] Bayesian calculation of probabilities that Family 8334 is affected (99.2%; CMD+) and unaffected (0.8%; CMD-) with *BRCA2*-related cancer predisposition when phenotype data from genotyped members of the family were incorporated. (S) and (T) were derived from the posterior probabilities (G) and (H), respectively, shown in Supplemental Methods Table 1. (U) is the value of (Q) from Supplemental Methods Table 2 and (V) is the value of (R) from Supplemental Methods Table 2. (W) is the product of (S) and (U) and (X) is the product of (T) and (V). Posterior probabilities in the last line of the table were derived per standard Bayesian arithmetic. Decimals are used for most probabilities, but percentages are used for the posteriors for clarity.

This result shows that the likelihood of a positive CMD for *BRCA2*-related cancer predisposition has jumped to nearly 100% based on the presence of breast cancer in two members of this family who harbor the variant. This fits with clinical intuition as such a family is more likely to have *BRCA2*-related cancer predisposition rather than having coincidental breast cancer unassociated with that disorder. That the proband is not currently affected with cancer does not much detract from this conclusion, which again fits with clinical intuition.

The next step that can be considered was to include relatives who have not been tested for the variant but are related to an individual who is positive for the variant. Their likelihood of

harboring the familial variant can be derived from Mendelian principles. If there were no tested individuals other than the proband, the likelihood that each of the parents harbored the variant is sufficiently close to 50% that this number can be used for these calculations (*de novo* variants in these genes occur in about 1% of simplex occurrences of these disorders.) The grandparents each have a 25% likelihood of harboring the variant. However, since the variant in this pedigree is known to be present in the mother and maternal grandmother of the proband, the probability in the proband's father and paternal grandparents falls to zero.

To use these individuals for the risk calculation we used the concept of marginal risk of disease. The example shown in below in Supplemental Methods Table 4 considers the two relatives who were relevant to the risk calculation (they may harbor the variant) but who are not genotyped, the brother and the maternal aunt of the proband. This 61-year-old woman has a 6.0% chance of having cancer if she did not have *BRCA*2-related cancer predisposition and a 44.8% chance of having cancer if she did have *BRCA*2-related cancer predisposition. Having this condition incurs a marginal risk of cancer of 38.8% (the difference in her cancer risk at a given age with and without a risk-increasing cancer predisposition, data from ASK2ME). For an untested 61-year-old female in a family with causative *BRCA2* variant, her marginal cancer risk was the marginal risk for a known *BRCA2* heterozygote (38.8%) multiplied by the probability that she harbored a pathogenic *BRCA2* variant. If she is a first degree relative of an individual with that disorder but had herself not been tested for the variant, her marginal risk of cancer was attributable to her relatedness to a variant-positive individual is (0.5 x 0.388) = 0.194. We then added this risk back to her baseline risk of cancer (0.194 + 0.06) = 0.254 to derive her cancer risk at age 61. For a cancer-free, untested relative in our example pedigree, we used the inverse of her marginal risk of cancer (1.0 - 0.254 = 0.746).

Each of the untested relatives can be calculated similarly. For practical reasons, we limited this to first- and second-degree relatives unless there was a third or fourth degree relative who was positive for a relevant cancer. This is because the ratio of their probabilities for unaffected (probability for heterozygote/probability for population) is so close to 1.0 that it does not much affect the posterior probability of a CMD of the family. Note that individuals who were variant-negative in the cascade testing were not included as they were irrelevant to the calculations. In this family there were two relatives included in the calculations shown in Supplemental Methods Table 4 below. Because neither of these individuals had cancer, probabilities can be kept consistent (i.e., probability of having cancer or being cancer-free) throughout the table. Note that the maternal aunt was considered a first degree relative because she was the daughter of a test-positive individual in this family (maternal grandmother of the secondary finding recipient).

Supplemental Methods Table 4: Probability Calculation for Untested Relatives[a]

Relative, affection status	Degree of relation	Conditional Probability of being cancer-free given CMD-	Conditional Probability of being cancer-free given CMD+	Marginal conditional probability	Marginal conditional probability given relationship	Adjusted conditional probability for CMD+ considering

						likelihood of being variant +
35 yo Brother, unaffected by cancer	1 (A)	1.0 (B)	0.996 (C)	0.0044 (D)	0.0022 (E)	0.998 (F)
61 yo Maternal Aunt, unaffected by cancer	1	0.940	0.552	0.389	0.194	0.746
Joint probability		0.940 (G)	0.549 (H)			0.744 (I)

[a] (A) is the degree of relationship to a tested individual. (B) is the probability of this individual's observed clinical status assuming the family is CMD-. (C) is the probability of this individual's observed clinical status assuming that the family is CMD+. (D) is the difference between (B) and (C). (E) is the proportion of genes this individual shared with the tested individual ($0.5^{(A)}$; 50% in this case for the sister of the proband) multiplied by the individual's marginal risk (D). To derive (F), (C) was added to the product of (D) and the proportion of shared genes with a tested individual ($0.5^{(A)}$). (G), (H), and (I) are joint probabilities calculated per standard Bayseian arithmetic (multiplying probabilities in the same column).

Finally, we combined all available testing and clinical data to estimate the probability that family 8334 is affected with *BRCA2*-related cancer predisposition in Supplemental Methods Table 5.

Supplemental Methods Table 5: Bayesian Probability of CMD in a Person With an LP Variant, Taking All Available Data About Affection and Genotype Status into Account[a]

	Probability affected (CMD+)	Probability unaffected (CMD-)
Prior (from Supplemental Methods Table 3)	0.992 (A)	0.008 (B)
Conditional probability of health status (from Supplemental Methods Table 4)	0.744 (C)	0.940 (D)
Joint probability	0.738	0.008
Posterior Probability	98.9% (E)	1.1%

[a](A) and (B) were calculated based on the observed clinical and genotypic data from tested individuals in the family; they are the same as the posterior probabilities in Supplemental Methods Table 3. Conditional probabilities in the second row were derived from Supplemental Methods Table 4, the conditional probability that untested relatives are CMD+ (C) or CMD- (D) based on their observed health statuses and degree of relationship to a tested individual. (E) is the posterior probability that family 8334 had a postitive clinic-molecular diagnosis of *BRCA2*-related cancer predisposition incorporating all available data from the family.

Note that adding the two untested individuals to the calculation hardly changed the posterior probability of disease. This fits with clinical intuition in that it is not certain that they have the family variant and unaffecteds reduce the likelihood of a diagnosis of *BRCA2*-related cancer predisposition to a lesser degree than affecteds increase the likelihood of the diagnosis because of the incomplete penetrance of the disorder.

Supplemental Methods Table 6: Variations of Supplement Methods Table 1 Under Distinct Assumptions[a]

	A Baseline - merge P & LP	B Double disease frequency	C Halve disease frequency	D Reduce diagnostic yield	E Increase diagnostic yield	F Double false positive rate	G Halve false positive rate	H Consider Path variants separately	J Consider LP variants separately
Prior Prob Disease	0.0025	0.005	0.00125	0.0025	0.0025	0.0025	0.0025	0.0025	0.0025
P(T+|Disease+)	0.75	0.75	0.75	0.6	0.85	0.75	0.75	0.25	0.5
P(T+|Disease-)	0.001	0.001	0.001	0.001	0.001	0.002	0.0005	0.0001	0.0009
Jt+	0.001875	0.00375	0.0009375	0.0015	0.002125	0.001875	0.001875	0.000625	0.00125
Jt-	0.0009975	0.000995	0.00099875	0.0009975	0.0009975	0.001995	0.00049875	0.00009975	0.00089775
Post	65%	79%	48%	60%	68%	48%	79%	86%	58%

[a]Column A represents the baseline assumption in the beginning of the supplemental methods, which is that P & LP variants are considered together and the disease frequency is 1/400. The diagnostic yield is 75% and the false positive rate is 0.1%. Column B doubles the disease frequency (1/200). Column C halves the disease frequency (1/800). Column D reduces the diagnostic yield to 60%, compared to 75% in the baseline. Column E increases the diagnostic yield from 75% to 85%. Column F doubles the false positive rate from 1/1,000 to 1/500. Column G halves the false positive rate from 1/1,000 to 1/2,000. Column H considers pathogenic variants separately and column J considers likely pathogenic variants separately. Column J matches the posterior probabilities in Table 1 and was used as the starting point for the rest of the calculations shown in the prior tables.

Supplemental References

1. Katz, A.E., Nussbaum, R.L., Solomon, B.D., Rehm, H.L., Williams, M.S., and Biesecker, L.G. (2020). Management of Secondary Genomic Findings. Am J Hum Genet *107*, 3-14. 10.1016/j.ajhg.2020.05.002.
2. Petrucelli, N., Daly, M.B., and Feldman, G.L. (1993). BRCA1 and BRCA2 Hereditary Breast and Ovarian Cancer. In GeneReviews, R.A. Pagon, T.D. Bird, C.R. Dolan, K. Stephens, and M.P. Adam, eds.
3. Biesecker, L.G. (2019). Genomic screening and genomic diagnostic testing-two very different kettles of fish. Genome Med *11*, 75. 10.1186/s13073-019-0696-9.
